# Increased Rate of Incidental Colorectal Malignant Polyps: A Single-Center Experience

**DOI:** 10.1155/2018/3465931

**Published:** 2018-04-23

**Authors:** T. Buchbjerg, R. Kroijer, I. Al-Najami, K. Urth Hansen, G. Baatrup

**Affiliations:** ^1^Hospital South West Jutland, Esbjerg, Denmark; ^2^Department of Surgery, Odense University Hospital and Department of Clinical Science, University of Southern Denmark, Odense, Denmark

## Abstract

**Background and Aims:**

To investigate the incidence and treatment of colorectal malignant polyps before and after colorectal cancer screening initiation in March 2014 in a single Danish center.

**Materials and Methods:**

71 patients with colorectal malignant polyps in a single center from 2012 to 2015 were reported retrospectively.

**Results:**

There was a significant increase (*P* < 0.01) in the incidence of colorectal malignant polyps from 2012 to 2013 and 2014 to 2015 (8 versus 63) relative to the increase in colonoscopies with polypectomy (1029 versus 2706). It coincides with the initiation of screening in March 2014. A positive, nonradical, or undeterminable resection margin was found in 57% (36/63), and this was the primary indication for surgery. Additional surgery was done in 49% of the cases (31/63) with 27 bowel resections and 4 transanal endoscopic microsurgery (TEM) procedures. Nineteen percent (5/27) had either residual cancer cells at the polypectomy site or lymph node metastasis in the resection specimens.

**Conclusion:**

Colorectal malignant polyps have become more frequent after the initiation of screening. The primary, and operator-dependent, indicator for surgery is the positive, nonradical, or undeterminable resection margin, and 1 in 5 operated has remaining cancer in the resection specimens.

## 1. Introduction

Colorectal cancer (CRC) screening was initiated in Denmark in 2014. CRC screening is offered to all citizens aged 50 to 74 years, with primary immunofecal occult blood test and subsequent colonoscopy if tested positive [1]. The screening program aims to promote the finding of cancers at an earlier stage and removal of precursor colorectal adenomas. Polyps found during colonoscopy are routinely removed using snare polypectomy; however, larger and sessile or flat lesions are preferably removed by endoscopic mucosa resection (EMR), endoscopic submucosal dissection (ESD), or transanal endoscopic microsurgery (TEM). This is to ensure radical excision to reduce recurrence rates and because larger polyps are more likely to harbour cancer [2].

This study investigates the changes in endoscopic procedures performed before and after the CRC screening initiation with focus on polyp characteristics, management, treatment, and outcome of the treatment of incidental colorectal malignant polyps in a single center. We will primarily focus on the patients found in 2014-2015 where the incidence of incidental malignant polyps suddenly rose significantly compared with the years before CRC screening.

Furthermore, we report the findings of previously published papers on the subject.

## 2. Materials and Methods

We retrieved data from our database for endoscopic procedures and colorectal cancers from 1 January 2012 to 31 December 2015, and individuals with incidental malignant polyps were identified. Only polyps assessed clinically as benign, but where histology revealed adenocarcinoma, were included in the study. In equivocal cases, the specimen was reassessed by one dedicated pathologist. A literature search was done on incidental colorectal malignant polyps through MEDLINE with focus on the following results: the ratio of salvage surgical resections, the incidence of residual adenocarcinoma at the polypectomy site, and the lymph node status in those resected.

When encountering an incidental malignant polyp, the current Danish national guideline is as follows: patients undergo computed tomography (CT) of the thorax and abdomen in colonic lesions, in addition to pelvic magnetic resonance imaging (MRI) in case of incidental malignant polyps in the rectum defined as lesions up to 15 cm from the anal verge [3]. In our institution, the polypectomy site is marked by dye at reendoscopy once the cancer diagnosis is confirmed. The pathology report includes stratification of the cancer according to submucosal invasion (Haggitt's level for pedunculated polyps and Kikuchi sm grade for flat or sessile polyps) and an evaluation of the resection margin, lymphovascular invasion, and tumor differentiation [4]. Histological high-risk features indicating surgery are nonradical resection (adenocarcinoma ≤ 1 mm from the resection margin), adenocarcinoma in the resection margin, histologically unclear resection margin (piecemeal), lymphovascular invasion, Haggitt level 3-4, Kikuchi sm stage 2-3, and poor tumor differentiation. Patients with high-risk features are advised to undergo bowel resection for oncological safety due to the risk of lymph node metastasis [5–8]. Tumor budding is still controversial as a high-risk feature but is discussed at the multidisciplinary team (MDT) meeting where all patients are evaluated.

Statistical analyses were performed using STATA 14. A chi-square test was applied to ratios or proportions to assess the hypothesis of no difference in the outcome of the categorical data. *P* values < 0.05 were considered significant.

## 3. Results

The incidence of incidental malignant polyps at our center was significantly higher with screening in 2014-2015 compared with 2012-2013 without screening (*P* < 0.01) relative to the increased number of colonoscopies with polypectomy (Table 1). The 2014-2015 patient and polyp characteristics are shown in Table 2. Forty-six percent (29/63) of the polyps were excised in screening colonoscopies, the mean polyp size was 19 mm, and 92% were located in either the sigmoid colon or the rectum. Sixty patients were treated with simple snare polypectomy and 3 with EMR because of large but putatively benign polyps. All polypectomies were defined as macroscopically complete by the endoscopist. There was a tendency of polyps larger than 20 mm, having a higher proportion of piecemeal resections (*P*=0.055).

High-risk features were found in 60% (38/63) of the cases indicating the need for additional surgery. Twenty-two patients had undetermined resection margins due to piecemeal resection, fourteen patients had nonradical polypectomy resection margins, and two patients had Haggitt level 3. Two of these patients had lymphovascular invasion and nonradical resection margin. None had Haggitt level 4, Kikuchi sm stage 2-3, or poorly differentiated carcinoma.

**Figure 1:**
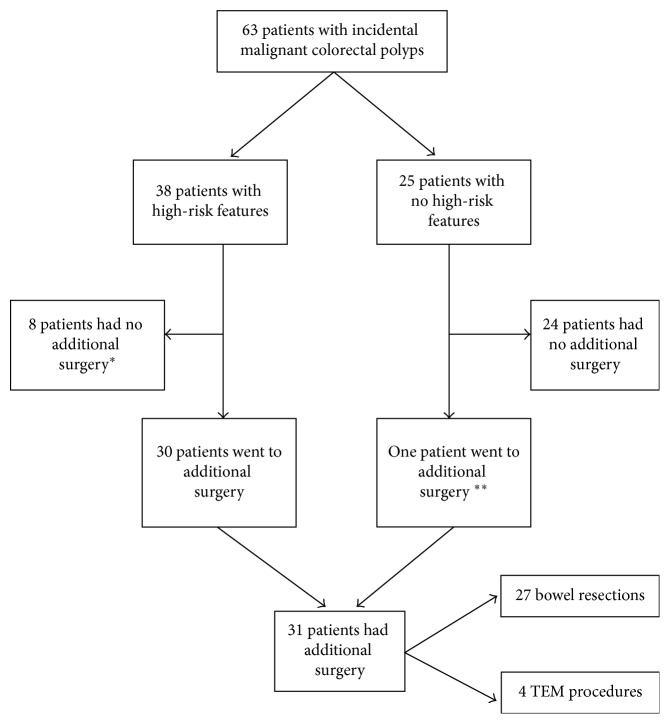
Flowchart showing the course of 63 patients with incidental colorectal malignant polyps. ^∗^Four rectal patients went to the surveillance program, three patients had too much comorbidity and one patient had radiation therapy. ^∗∗^The patient was referred to additional surgery because of suspicious lymph nodes on CT.

As demonstrated in the flowchart (Figure 1), major salvage surgery was performed in 43% (27/63) of the patients and local completion excision as TEM was done in an additional 4 patients. The reason for surgery as either major surgery or TEM was predominantly nonradical or undeterminable resection margins constituting 90% (28/31). In addition, one patient received major surgery due to a suspected malignant lymph node on CT and two due to Haggitt level 3.

In the salvage surgery group, 7.4% (2/27) had residual adenocarcinoma at the polypectomy site, 11.1% (3/27) had N1 disease, and none had both. In total, 19% (5/27) had either residual cancer at the polypectomy site or N1 disease in the salvage surgery group.

Furthermore, there were 4 patients with malignant rectal polyps that underwent subsequent TEM due to undeterminable resection margins in three cases and one with less than 1 mm to the resection margin. Of these, 25% (1/4) had residual adenocarcinoma in the TEM specimen. This patient had radical resection margins in the TEM specimen and no other high-risk features and therefore went to the surveillance program.

The complication rate in the surgery group was 11% (3/27) with two anastomotic leaks and one patient with perioperatively treated ureteral lesion, which was repaired laparoscopically, and there were no postoperative complications for this patient. This means that 7% (2/27) of the operated patients had a clinically severe complication. There was no 30-day mortality.

### 3.1. Literature Search Results

Table 3 shows the results of similar studies. The majority are retrospective and report rates of residual adenocarcinoma at the polypectomy site from 2 to 23% [8–11, 14, 15]. Two studies [12, 13] stand out with 32–58% residual adenocarcinoma at the polypectomy site. The rates of nodal metastasis vary from 3 to 17% [9–13, 15], but also in these cases, one study [14] found a higher proportion of patients with lymph node metastasis of 32%.

## 4. Discussion

A significant increase in malignant polyp incidence after the onset of screening was observed, but only 46% of these were found in CRC screening. We suspect that public and general practitioner awareness of bowel symptoms increased in parallel to the onset of screening. Leading up to the initiation of the CRC screening in Denmark, there was a national television campaign regarding bowel symptoms and colonoscopy which led to increased awareness on this topic.

Polyp localization was primarily in the sigmoid and rectum constituting >90% which is supported by the findings of other studies [2, 10, 13].

Thirty-eight patients had high-risk features and were all offered surgery, but Figure 1 shows that not all patients had their surgery done. The primary indication for completion of surgery (resection or TEM) was not radical polypectomy resection margin (28/31) as either positive, nonradical (≤1 mm free margin), or undeterminable resection margin.

We found an incidence of residual adenocarcinoma at the polypectomy site after salvage surgery of 7% (2/27) which is in concordance with previously reported rates of 2–18% (Table 3). Two studies [12, 13] have found 32–58% of their cohorts to have residual adenocarcinoma in the surgical specimens, but these studies have a smaller sample size compared to the other studies in Table 3, which may explain the high rate. Naqvi et al. [12] has the highest rate of residual adenocarcinoma of 58% (7/12), most likely because their sole indication for surgery was adenocarcinoma in the margin after polypectomy. No patients in their study had less than 1 mm to the resection margin or undetermined margins due to piecemeal polypectomy, which for most of the studies is the predominant indication for surgery. This could explain the high rate of residual adenocarcinoma in this study.

We found the incidence of lymph node metastasis to be 11% (3/27) which is consistent with the report of other studies (3–17%) (Table 3). Gonçalves et al. [14] found a higher rate of N1 disease of 32% (10/31). This high N1 rate may be due to the fact that none of the patients in this study had simple snare polypectomy of a simple pedunculated polyp. All their patients were found after EMR indicating that these patients had large, flat, or sessile polyps and thereby more complex polyps than simple pedunculated polyps. The EMR technique also has a higher risk of piecemeal resection. This results in 71% (22/31) being surgically resected due to the undeterminable resection margin in this study [14].

Even though we used national guidelines for pathology high-risk factors to decide which treatment to choose, our results show that 81% (22/27) had a major operation with no residual malignancy in the specimen. By far, the most frequent high-risk feature was not radical or uncertain resection margin, but only 7% (2/27) had residual malignancy at the polypectomy site after surgical resection. Of course, this high-risk feature is also important in regard to lymph node metastasis which is supported by our findings that 3 patients without residual malignancy at the polyp site actually had N1 disease. The high rate of negative pathology specimens after salvage surgery for incidental polyp malignancy is problematic, but it is also what other studies have found [8, 9, 10, 11, 15, 16].

All of the studies in Table 3, apart from Naqvi et al. [12], have the same indicators for surgery as used in our center and are also retrospective studies with 50–143 patients included. Standing out, Jung et al. [15] suggest that patients with Kikuchi sm stage 2 should undergo oncological resection in spite of >1 mm free resection margin. This is due to one case in their cohort who underwent oncological surgery, and the pathology report revealed N1 disease. This is debatable, and patients should always be discussed individually on MDT meeting for tailored treatment.

The primary reason for additional surgery as either major surgery or TEM in our study was undetermined or positive resection margin, which is also the only operator-dependent risk factor. Several other studies also found this to be the primary indication for salvage surgery [9, 10, 15]. Our results confirm the known risk of large or sessile polyps not having radical resection margins and indicate that when dealing with large, sessile, or rectal polyps, the polypectomy should be done with special attention to the resection margin. No patients in our data had Haggitt level 4 invasion or Kikuchi sm stage 3, which is probably due to many nonradical polypectomies making an exact Kikuchi and Haggitt level staging uncertain.

When dealing with malignant polyps in the rectum, our study indicates that the surgeon and the patient are more likely to select other treatments than major surgery in spite of high-risk features such as undeterminable resection margin. In our study, only 38% (5/13) had major surgery meaning that 62% (8/13) had other minimally invasive treatments or were followed up in the surveillance program. This is probably due to the higher risk of complications and the frequency of postoperative morbidity in major rectal surgery compared to colonic resections, such as low anterior resection syndrome, genitourinary dysfunction, and permanent stoma. The rectum should be considered differently than the colon in the treatment approach due to a wider range of treatment possibilities such as TEM or radiotherapy. The first intervention should allow options for salvage surgical treatment or complete local excision in cases of undiagnosed adenocarcinoma and should minimize the risk of potentially compromising a curative radical resection if deemed necessary.

Limitations of this study are the retrospective design and the low number of patients, which could result in reporting and selection bias.

## 5. Conclusion

After the introduction of colorectal cancer screening, the incidence of incidental malignant polyps has significantly increased in our centre. Since only 46% of these patients were found from screening colonoscopies, the reason for this is unknown, but we suspect that a combination of CRC screening initiation and an increase in the general awareness of bowel symptoms could be the reason.

As other studies have shown, we found a high rate of patients of 81% with no residual malignancy in the surgical specimen, which is problematic because these patients have gone through an unnecessary operation and risk of severe complications. This is always a risk with these patients, but nonetheless, this outlines the urgent need for high-level large studies so that more efficient guidelines can be made and help choosing the right treatment for patients with incidental colorectal polyp malignancy. It is probably not possible to reach 100% certainty that no patient will receive surgery without residual malignancy in the specimen, but the aim must be to reach better criteria for selecting patients for either salvage surgery or surveillance and thereby minimizing the risk of patients undergoing unnecessary surgery. In this study, the rate of residual malignancy after major surgery was 19%.

The results here underline the need for national and international guidelines including guidelines for polyp referral to advanced polypectomy such as EMR, ESD, or TEM, as well as recommendations for treatment and follow-up. Furthermore, the frequency of residual cancer among the patients treated with additional surgery and recurrence rates among the endoscopically treated patients without additional surgery should be weighed against the operative risks in trials with a longer follow-up period. Large-scale studies with long follow-up periods are needed to further investigate which treatment is preferable for incidental colorectal malignant polyps.

We will of course follow our patients in the future and evaluate their long-term outcomes.

## Figures and Tables

**Table 1:** Incidence of incidental colorectal malignant polyps relative to polypectomies in two time periods, before and after the onset of colorectal cancer screening.

Period	2012-2013	2014-2015	
Colonoscopies, *N*	4723	9333	—
Polypectomies^∗^, *N*	1029	2706	*P*=0.002
Malignant polyps	8	63	
Malignant polyps per 100 polypectomies	0.8	2.3	

^∗^Colonoscopies with at least one polypectomy.

**Table 2:** 2014 and 2015 demographics and polyp characteristics.

Total number of patients, *N*	63
Sex, *N* (%)	
Male	32 (51%)
Female	31 (49%)
Age (years)	
Median	68
Range	42–87
Reason for referral, *N* (%)	
Screening	29 (46%)
Others	34 (54%)
Polyp location, *N* (%)	
Right colon	3 (5%)
Transverse colon	1 (1.5%)
Descending colon	1 (1.5%)
Sigmoid colon	34 (54%)
Rectum	24 (38%)
Polyp size (mm)	
Mean	19
Range	6–45
<10 mm	10 (16%)
10–19 mm	29 (46%)
>19 mm	24 (38%)
Polyp morphology, *N* (%)	
Pedunculated	40 (63%)
Sessile	15 (24%)
Flat	0 (0%)
Not reported	8 (13%)
Piecemeal resection, *N* (%)	
Yes	22 (35%)
No	41 (65%)
Resection margin	
Radical (margin > 1 mm)	27 (43%)
Not radical (margin ≤ 1 mm)	36 (57%)

The polyp size was assessed by the pathologist.

**Table 3:** Chronologically listed results of studies reporting the rate of residual adenocarcinoma at the polypectomy site and lymph node metastasis after surgical resection following endoscopically resected colorectal incidental malignant polyps.

Article	Year	*N*	Patients per year, *N*	Number of surgically resected patients, *N*	Residual adenocarcinoma at the polypectomy site, % (95% CI)	Lymph node metastasis, % (95% CI)	Either residual cancer or lymph node metastasis, % (95% CI)
Choi et al. [8]	2008	168	11	168	14% (8.8–19.3)	n.a.	n.a.
Boenicke et al. [9]	2009	105	7	65	2% (0–5.4)	11% (3.4–18.6)	n.a.
Butte et al. [10]	2011	143	8	143	7% (2.8–11.2)	10% (5.1–15.0)	n.a.
Benizri et al. [11]	2012	64	6	64	3% (0–7.2)	8% (1.4–14.7)	n.a.
Naqvi et al. [12]^∗^	2012	65	6	12	58% (30.1–86.0)	17% (0–38.3)	n.a.
Steigen et al. [13]^∗^	2013	74	6	19	32% (11.0–53.0)	5% (0–14.9)	n.a.
Gonçalves et al. [14]	2013	40	8	31	23% (8.2–37.9)	32% (15.6–48.4)	48% (30.4–65.6)
Jung et al. [15]	2015	50	12	50	18% (7.4–28.7)	10% (1.7–18.3)	18% (7.6–28.7)
Levic et al. [16]^∗^	2015	50	10	23	22% (5.1–39.0)	22% (5.1–39.0)	30% (11.3–48.8)
Current study	2016	63	31	27	10% (0–20.5)	11% (0–22.3)	19% (4.2–33.9)

^∗^Studies with less than 25 patients undergoing additional major surgical resection; 95% CI: 95% confidence interval; n.a.: not available.
